# The influence of marital status at diagnosis on survival of adult patients with mantle cell lymphoma

**DOI:** 10.1007/s00432-024-05647-z

**Published:** 2024-03-11

**Authors:** Ting Zhang, Zhao-tong Wang, Zhuo Li, Shuo-xin Yin, Xun Wang, Hai-zhu Chen

**Affiliations:** 1 Department of Oncology, Nanyang Central Hospital, Nanyang, People’s Republic of China; 2 Department of Psychiatry, Jinling Hospital, Medical School of Nanjing University, Nanjing, People’s Republic of China; 3 Guangdong Provincial Key Laboratory of Malignant Tumor Epigenetics and Gene Regulation, Breast Tumor Centre, Department of Medical Oncology, Phase I Clinical Trial Centre, Sun Yat-sen Memorial Hospital, Sun Yat-sen University, Guangzhou, People’s Republic of China

**Keywords:** Mantle cell lymphoma, Marital status, SEER, Survival analysis

## Abstract

**Purpose:**

Marital status has been reported to influence the survival outcomes of various cancers, but its impact on patients with mantle cell lymphoma (MCL) remains unclear. This study aimed to assess the influence of marital status at diagnosis on overall survival (OS) and cancer-specific survival (CSS) in patients with MCL.

**Methods:**

The study utilized data from the National Cancer Institute’s Surveillance, Epidemiology, and End Results (SEER)-18 databases, including 6437 eligible individuals diagnosed with MCL from 2000 to 2018. A 1:1 propensity matching method (PSM) minimized confounding factor. Univariate and multivariate analyses determined hazard ratios (HR). Stratified hazard models were developed for married and unmarried statuses across time intervals.

**Results:**

Married patients exhibited better 5-year OS and CSS rates compared to unmarried patients (54.2% vs. 39.7%, log-rank *p* < 0.001; 62.6% vs. 49.3%, log-rank *p* < 0.001). Multivariate analysis indicated that being unmarried was an independent risk factor for OS (adjusted HR 1.420, 95% CI 1.329–1.517) and CSS (adjusted HR 1.388, 95% CI 1.286–1.498). After PSM, being unmarried remained an independent risk factor for both OS and CSS. Among unmarried patients, widowed individuals exhibited the poorest survival outcomes compared to patients with other marital statuses, with 5-year OS and CSS rates of 28.5% and 41.0%, respectively. Furthermore, in the 10-year OS and CSS hazard model for widowed individuals had a significantly higher risk of mortality, with the probability of overall and cancer-specific mortality increased by 1.7-fold and 1.6-fold, respectively.

**Conclusion:**

Marital status at diagnosis is an independent prognostic factor for MCL patients, with widowed individuals showing worse OS and CSS than those who are married, single, or divorced/separated. Adequate psychological and social support for widowed patients is crucial for improving outcomes in this patient population.

**Supplementary Information:**

The online version contains supplementary material available at 10.1007/s00432-024-05647-z.

## Introduction

Mantle cell lymphoma (MCL) was identified as a specific type of lymphoma in 1992 (Banks et al. 1992). It is a rare subtype of aggressive B cell non-Hodgkin lymphoma (NHL), accounting for 3% to 10% of adult NHL, the incidence is on the rise, with a median age at diagnosis of 68 years and a male-to-female ratio of 2.3–2.5:1 (Abrahamsson et al. 2014). Approximately 1 in 200,000 individuals per year are diagnosed with MCL, and in the United States, the incidence is approximately 4 to 8 cases per million persons per year (Teras et al. 2016). Patients with MCL usually present with enlarged lymph nodes at multiple sites, the majority of patients are diagnosed with advanced disease, and CyclinDl expression is characteristic (Jain and Wang 2019). Most patients do not respond well to chemotherapy and have a poor prognosis, with a median survival of 3–4 years (Jain et al. 2022).

The study found that social factors such as marital status, race, education, income, and occupation were associated with cancer mortality (Hemminki et al. 2003; Hashibe et al. 2011; Lortet-Tieulent et al. 2020). Many studies have shown that marital status is associated with the prognosis of many cancers, including lung cancer (Wu et al. 2022), gastric cancer (Jin et al. 2016), colorectal cancer (Li et al. 2015), mycosis fungoides (Xing et al. 2021), and Hodgkin's lymphoma (Wang et al. 2017).

Although many studies have confirmed the relationship between marital status and the survival of cancer patients. So far, no study has shown the impact of marital status on the survival outcomes of MCL patients. Therefore, this study explored the impact of marital status at diagnosis on the overall survival (OS) and cancer-specific survival (CSS) of MCL patients by analyzing data from the Surveillance, Epidemiology, and End Results (SEER) database.

## Materials and methods

### Data source

The Surveillance, Epidemiology, and End Results (SEER) Program of the National Cancer Institute is a publicly available and reliable cancer database. We screened data on 13,105 MCL patients from the SEER-18 Registries, November 2020 Submission (2000–2018), using SEER * Stat software (version 8.4.2 released on 8/14/2023).

SEER-18 database contains cancer data from 18 SEER registries, including: San Francisco-Oakland SMSA, Connecticut, Detroit (Metropolitan), Hawaii, Iowa, New Mexico, Seattle (Puget Sound), Utah, Atlanta (Metropolitan), San Jose-Monterey. Los Angeles, Alaska Natives, Rural Georgia, California excluding SF/SJM/LA, Kentucky, Louisiana, New Jersey, Greater Georgia. It encompasses approximately 27.8% of the U.S. population (National Cancer Institute 2023).

**Fig. 1:**
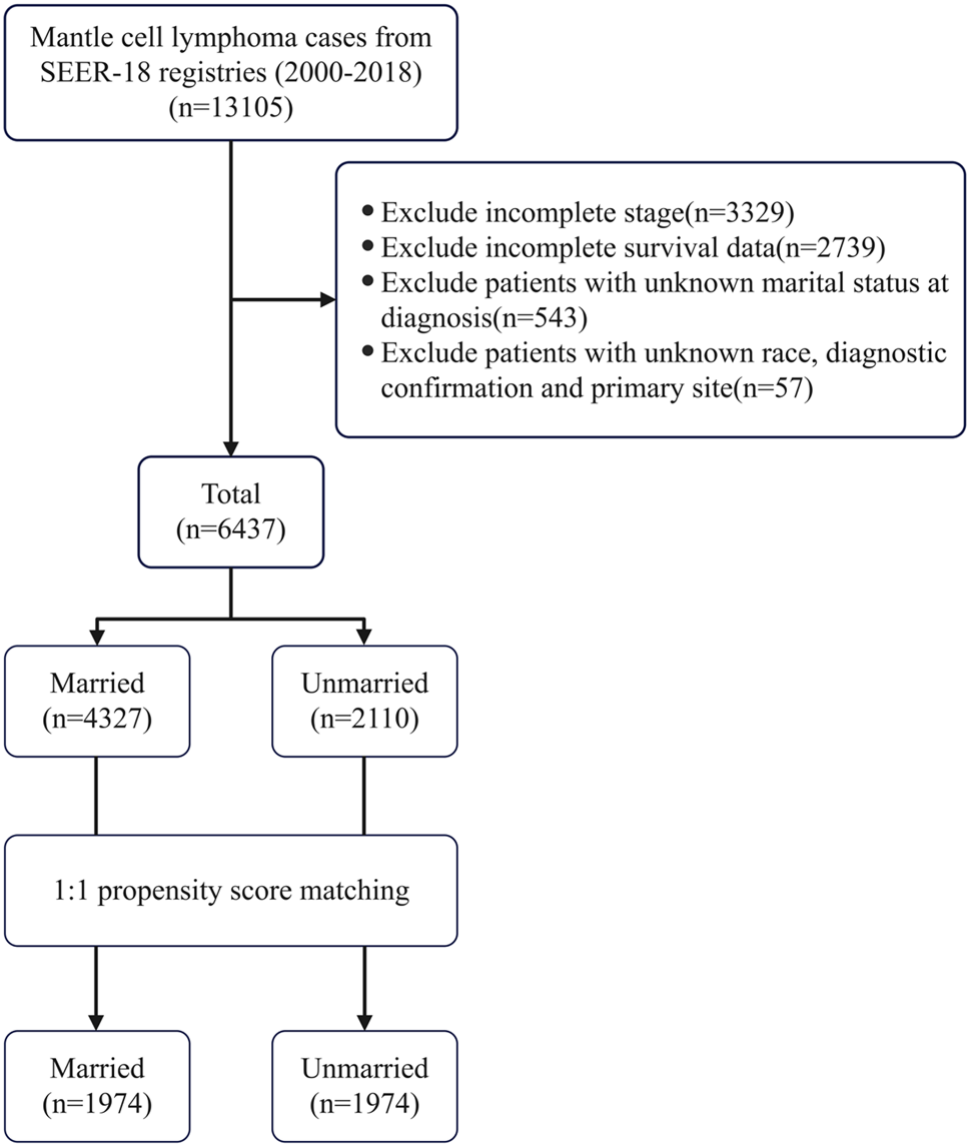
Flow diagram of data process for mantle cell lymphoma patients

The diagnosis of all MCL patients was confirmed by the International Classification of Diseases for Oncology, third edition (ICD-O-3) histology code 9673/3. The exclusion criteria are as follows: (1) patients with incomplete Ann Arbor stage or missing/incomplete survival data and follow-up, (2) unknown marital status at diagnosis or unknown race, (3) unknown diagnostic confirmation or not first primary site. Based on the above exclusion criteria, 6437 eligible patients were enrolled in the study (Fig. 1). This was a retrospective study, analyzing data from the SEER public database; therefore, ethical approval was not required.

### Demographic and clinical variables

Patient demographic variables included sex, age, race, and marital status at diagnosis. Clinical variables included treatment information (chemotherapy, and radiotherapy), primary site, Ann Arbor stage, survival time, survival status, and causes of death. Age categories were delineated as < 50, 50–59, 60–69, and ≥ 70, according to the MIPI age classification (Hoster et al. 2008). Race/ethnicity classifications comprised Hispanic Non-Hispanic White, Non-Hispanic Black, and Other (encompassing American Indian, Alaska Native, Asian, and Pacific Islander categories). MCL staging adhered to the Lugano staging system (Yoo 2022), distinguishing between Limited (stages I and II) and advanced diseases (stages III and IV). The year of diagnosis was stratified into three periods: 2000–2006, 2007–2010, and 2011–2015.

### Study endpoints

The endpoints of the study included OS and CSS. OS was defined as the time from the start of the first diagnosis or treatment to the time when the patient died or was last followed up for any cause. CSS was defined as the time from the start of the first diagnosis or treatment to the time when the patient died or was last followed up for MCL-related causes.

### Statistical analysis

The chi-square test was used to compare the categorical variables of clinical characteristics in each group, Age and survival time were presented using the median and interquartile range (IQR), while descriptive statistics for continuous variables were expressed in terms of mean and standard deviation. the Kaplan–Meier method was used to calculate the survival rate and construct a survival curve, and the Log-rank test was used for comparison between groups. Cox proportional hazards regression models were used for univariate and multivariate analysis and the hazard ratio [HR] between variable and mortality was calculated. All confidence intervals (CI) are stated with a 95% confidence level.

A propensity matching method (PSM) was employed to minimize potential confounding factors in studies, thereby equalizing differences in clinical characteristics between groups. In this study, a 1:1 nearest-neighbor matching method was applied to marital status, with a caliper value set at 0.01 for matching tolerance. Additionally, we constructed stratified hazard models for unmarried status across different time intervals (1-, 5-, and 10-year), calculating the HR to delineate the associations between various unmarried statuses and the probability of mortality.

All data were analyzed using IBM SPSS statistical software version 26.0 and R, version 4.2.1 (http://www.r-project.org/). A two-sided *p*-value < 0.05 was considered statistically significant.

## Results

### Clinical characteristics of the patient

From 2000 to 2018, 6437 eligible MCL patients were analyzed using the SEER-18 Database. Among them, 4327 (67.2%) were identified as married, while 2110 (32.8%) were categorized as unmarried. The median age of the entire cohort was 68 years (IQR 59–76 years). Notably, the unmarried group exhibited a median age of 70 years (IQR 59–79 years), whereas the married group had a median age of 67 years (IQR 59–75 years). Approximately 45.2%/6437 of the total patients were aged ≥ 70 years, with a higher proportion in the unmarried group (50.3%). The median survival time for the entire cohort was 47 months (IQR 118–85 months), with the married group having a median survival time of 52 months (IQR 22–91 months) and the unmarried group having 38 months (IQR 13–70 months). Statistically significant differences were observed in sex (*p* < 0.001), age (*p* < 0.001), race/ethnicity (*p* < 0.001), stage (*p* = 0.007), sequence number (*p* < 0.001), chemotherapy (*p* < 0.001), and radiation (*p* = 0.036), while year of diagnosis and primary site did not show significant differences.

To address potential biases, a 1:1 PSM was conducted, resulting in a cohort of 3948 MCL patients, evenly split between married and unmarried groups. Except for the year of diagnosis, all other variables, including sex, age, race/ethnicity, stage, primary site, sequence number, chemotherapy, radiation, showed no significant differences (all *p* > 0.05), demonstrating good balance. The baseline clinical characteristics of MCL patients with different marital status are summarized in Table 1.

**Table 1:** Baseline clinical characteristics of patients with mantle cell lymphoma in the data before and after PSM

Characteristics	Data before PSM				Data after PSM			
	Overall (*N* = 6437) no. (%)	Married (*N* = 4327) no. (%)	Unmarried (*N* = 2110) no. (%)	*p*-value	Overall (*N* = 3948) no. (%)	Married (*N* = 1974) no. (%)	Unmarried (*N* = 1974) no. (%)	*p*-value
Sex				< 0.001				0.625
Male	4551 (70.7)	3330 (77.0)	1221 (57.9)		2403 (60.9)	1194 (60.5)	1209 (61.2)	
Female	1886 (29.3)	997 (23.0)	889 (42.1)		1545 (39.1)	780 (39.5)	765 (38.8)	
Age (year)				< 0.001				0.051
Median (IQR)	68 (59–76)	67 (59–75)	70 (59–79)		69 (58–77)	68 (58–75)	69 (58–78)	
Mean ± SD	67.44 ± 11.73	66.88 ± 11.05	68.58 ± 12.93		67.63 ± 12.15	66.92 ± 11.42	68.33 ± 12.80	
≤ 50	446 (6.9)	277 (6.4)	169 (8.0)		279 (7.1)	129 (6.5)	150 (7.6)	
50–59	1228 (19.1)	841 (19.4)	387 (18.3)		827 (20.9)	447 (22.6)	380 (19.3)	
60–69	1851 (28.8)	1359 (31.4)	492 (23.3)		955 (24.2)	473 (24.0)	482 (24.4)	
≥ 70	2912 (45.2)	1850 (42.8)	1062 (50.3)		1887 (47.8)	925 (46.9)	962 (48.7)	
Year of diagnosis				0.457				0.047
2000–2004	1399 (21.7)	929 (21.5)	470 (22.3)		840 (21.3)	412 (20.9)	428 (21.7)	
2005–2009	2023 (31.4)	1381 (31.9)	642 (30.4)		1345 (34.1)	737 (37.3)	608 (30.8)	
2010–2015	3015 (46.8)	2017 (46.6)	998 (47.3)		1763 (44.7)	825 (41.8)	938 (47.5)	
Race/ethnicity				< 0.001				0.450
Hispanic	464 (7.2)	277 (6.4)	187 (8.9)		335 (8.5)	159 (8.1)	176 (8.9)	
Non-Hispanic White	5385 (83.7)	3699 (85.5)	1686 (79.9)		3213 (81.4)	1603 (81.2)	1610 (81.6)	
Non-Hispanic Black	321 (5.0)	156 (3.6)	165 (7.8)		243 (6.2)	126 (6.4)	117 (5.9)	
Other	267 (4.1)	195 (4.5)	72 (3.4)		157 (4.0)	86 (4.4)	71 (3.6)	
Stage				0.007				0.865
Limited	1153 (17.9)	814 (18.8)	339 (16.1)		660 (16.7)	332 (16.8)	328 (16.6)	
Advanced	5284 (82.1)	3513 (81.2)	1771 (83.9)		3288 (83.3)	1642 (83.2)	1646 (83.4)	
Primary site				0.870				0.074
Nodal	5350 (83.1)	3594 (83.1)	1756 (83.2)		3321 (84.1)	1681 (85.2)	1640 (83.1)	
Extranodal	1087 (16.9)	733 (16.9)	354 (16.8)		627 (15.9)	293 (14.8)	334 (16.9)	
Sequence number				< 0.001				0.179
One primary only	4239 (65.9)	2769 (64.0)	1470 (69.7)		2731 (69.2)	1385 (70.2)	1346 (68.2)	
First of two or more	2198 (34.1)	1558 (36.0)	640 (30.3)		1217 (30.8)	589 (29.8)	628 (31.8)	
Chemotherapy				< 0.001				0.097
Yes	4931 (76.6)	3385 (78.2)	1546 (73.3)		2969 (75.2)	1507 (76.3)	1462 (74.1)	
No	1506 (23.4)	942 (21.8)	564 (26.7)		979 (24.8)	467 (23.7)	512 (25.9)	
Radiation				0.036				0.524
Yes	491 (7.6)	351 (8.1)	140 (6.6)		264 (6.7)	127 (6.4)	137 (6.9)	
No	5946 (92.4)	3976 (91.9)	1970 (93.4)		3684 (93.3)	1847 (93.6)	1837 (93.1)	
Survival time (months)								
Median (IQR)	47 (18–85)	52 (22–91)	38 (13–70)		45 (16–82)	51 (20–90)	39 (13–72)	
Mean ± SD	58.55 ± 49.11	62.64 ± 49.86	50.17 ± 46.45		56.12 ± 48.17	61.53 ± 49.25	50.70 ± 46.45	

PSM, propensity score matching; no, number; IQR, interquartile range; SD, standard deviatio

### Effects of marital status on OS and CSS in patients with MCL

The median OS and CSS of the whole population were 59 months (95% CI 56–62 months) and 89 months (95% CI 83–95 months), respectively. The 5-year OS and CSS rates were 49.4% (95% CI 48.2–50.7%) and 58.3% (95% CI 57.0–59.6%), respectively.

**Fig. 2:**
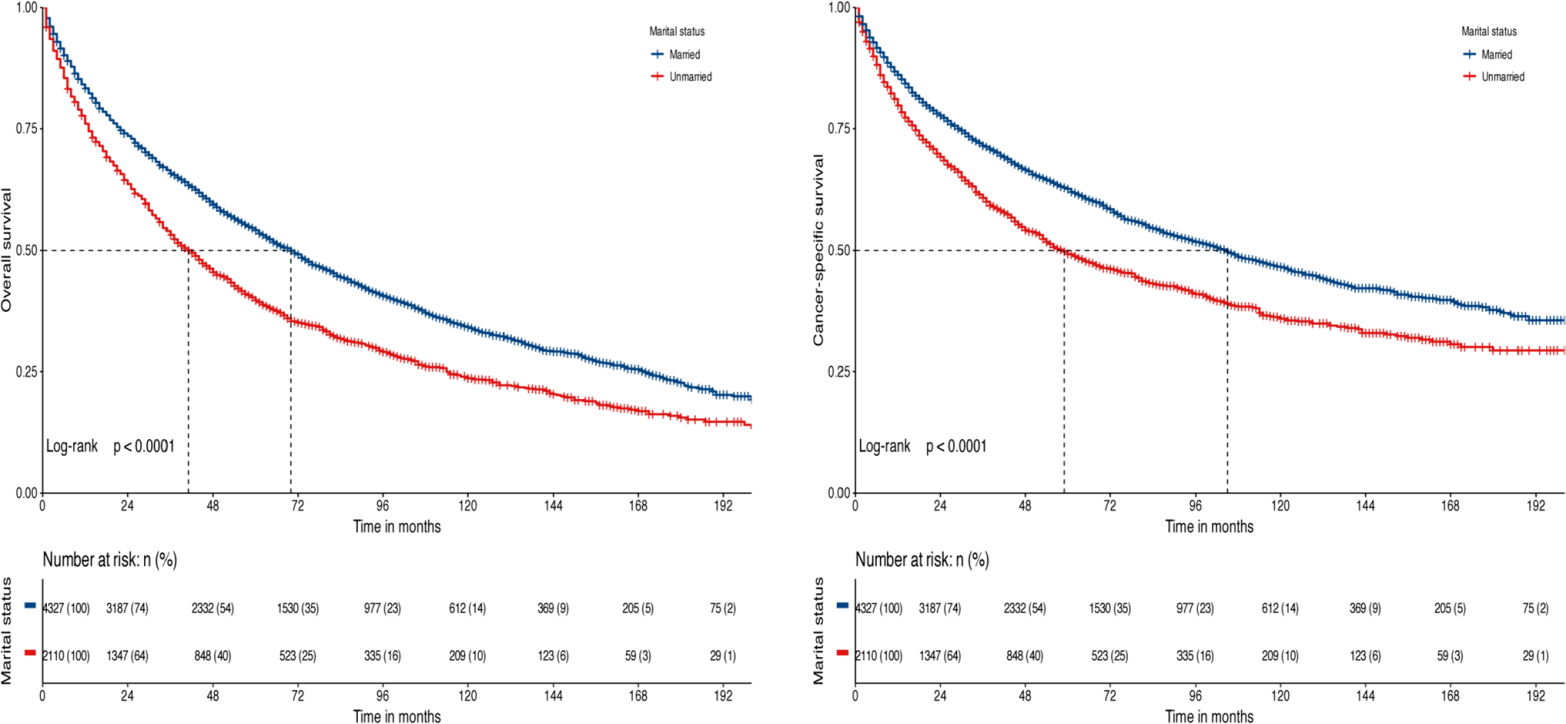
Kaplan–Meier curves present the overall survival and cancer-specific survival of patients with mantle cell lymphoma stratified by marital status

The 5-year OS rates for married and unmarried patients were 54.2% (95% CI 52.7–55.7%) and 39.7% (95% CI 37.6–41.9%), respectively (log-rank *p* < 0.001). The median OS for the two groups was 70 months (95% CI 66–74 months) and 41 months (95% CI 38–45 months), respectively. Similarly, the 5-year CSS rates for married and unmarried patients were 62.6% (95% CI 61.1–64.1%) and 49.3% (95% CI 47.0–51.6%), respectively (log-rank *p* < 0.001). The median CSS for the two groups was 105 months (95% CI 97–112 months) and 59 months (95% CI 54–66 months), respectively, as shown in Fig. 2.

**Fig. 3:**
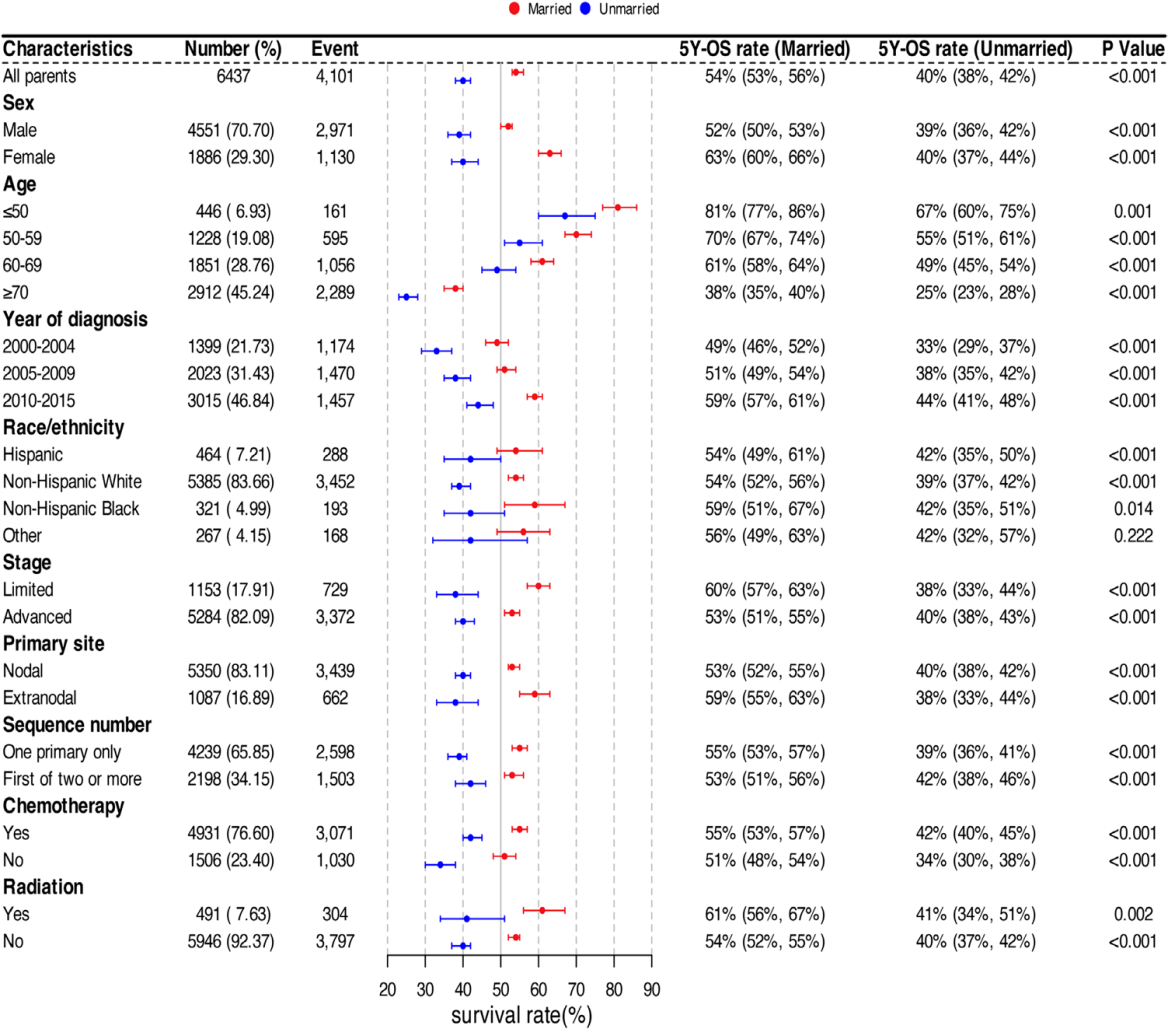
The 5-year overall survival rates among different marital statuses within each subgroup. 5Y-OS 5-year overall survival

Additionally, by analyzing the 5-year OS rates within different subgroups based on marital status, we found that except for individuals of other race/ethnicity* (p* = 0.222), there were significant differences in the 5-year OS rates among the remaining subgroups (*p* < 0.05). Particularly, the 5-year OS rate was at its lowest in elderly patients aged ≥ 70 years (married 38% vs. unmarried 25%), while the highest 5-year OS rate was observed in patients aged ≤ 50 years (married 81% vs. unmarried 67%) (Fig. 3). A similar trend was observed for the 5-year CSS rates in different subgroups based on marital status (Supplementary Fig. S1).

### Univariate and multivariate analysis of OS and CSS in patients with MCL

Univariate and multivariate Cox proportional hazards regression analyses were conducted to identify prognostic risk factors for MCL patients, with the results presented in Table 2. In univariate analysis, besides race/ethnicity, sequence number, sex, age, year of diagnosis, stage, primary site, chemotherapy, radiation, and marital status were found to be significantly associated with OS (*p* < 0.05). However, race/ethnicity, chemotherapy, and radiation did not show significant differences in CSS (*p* > 0.05). In multivariate analysis adjusting for confounding variables, unmarried status emerged as an independent risk factor for both OS and CSS, with HR of 1.420 (95% CI 1.329–1.517) and 1.388 (95% CI 1.286–1.498), respectively. Additionally, age categories 50–59 (HR 1.611, 95% CI 1.353–1.918, *p* < 0.001), 60–69 (HR 2.283, 95% CI 1.932–2.697, *p* < 0.001), and ≥ 70 years (HR 4.391, 95% CI 3.735–5.163, *p* < 0.001), advanced stage (HR 1.287, 95% CI 1.182–1.401, *p* < 0.001) were also independent prognostic factors significantly associated with worse OS. Moreover, these factors were also significantly correlated with worse CSS.

**Table 2:** Univariate and multivariate analyses of overall survival and cancer-specific survival in patients with mantle cell lymphoma

Variables	Overall survival				Cancer-specific survival			
	Univariate analysis	*p*-value	Multivariate analysis	*p*-value	Univariate analysis	*p*-value	Multivariate analysis	*p*-value
	HR (95% CI)		HR (95% CI)		HR (95% CI)		HR (95% CI)	
Sex								
Male	Reference		Reference		Reference		Reference	
Female	0.868 (0.810–0.929)	< 0.001	0.7242 (0.691–0.796)	< 0.001	0.843 (0.778–0.913)	< 0.001	0.853 (0.770–0.945)	0.002
Age (year)								
≤ 50	Reference		Reference		Reference		Reference	
50–59	1.538 (1.292–1.831)	< 0.001	1.611 (1.353–1.918)	< 0.001	1.370 (1.135–1.654)	0.001	1.483 (1.229–1.791)	< 0.001
60–69	2.094 (1.774–2.783)	< 0.001	2.283 (1.932–2.697)	< 0.001	1.727 (1.443–2.066)	< 0.001	2.016 (1.683–2.415)	< 0.001
≥ 70	4.073 (3.468–4.784)	< 0.001	4.391 (3.735–5.163)	< 0.001	3.059 (2.572–3.637)	< 0.001	3.666 (3.078–4.368)	< 0.001
Year of diagnosis								
2000–2004	Reference		Reference		Reference		Reference	
2005–2009	0.905 (0.837–0.979)	0.013	0.880 (0.813–0.952)	0.001	0.879 (0.803–0.962)	0.005	0.841 (0.768–0.920)	< 0.001
2010–2015	0.758 (0.700–0.822)	< 0.001	0.722 (0.666–0.783)	< 0.001	0.710 (0.647–0.778)	< 0.001	0.665 (0.606–0.729)	< 0.001
Race/ethnicity								
Hispanic	Reference				Reference		Reference	
Non-Hispanic White	1.006 (0.892–1.135)	0.919			0.888 (0.778–1.013)	0.078		
Non-Hispanic Black	0.939 (0.782–1.127)	0.497			0.812 (0.658–1.001)	0.051		
Other	0.972 (0.803–1.175)	0.766			0.947 (0.767–1.168)	0.610		
Stage								
Limited	Reference		Reference		Reference		Reference	
Advanced	1.177 (1.086–1.275)	< 0.001	1.287 (1.182–1.401)	< 0.001	1.381 (1.251–1.525)	< 0.001	1.472 (1.328–1.632)	< 0.001
Primary Site								
Extranodal	Reference				Reference		Reference	
Nodal	1.106 (1.017–1.201)	0.018	0.924 (0.849–1.006)	0.070	1.242 (1.123–1.373)	< 0.001	1.173 (1.058-.299)	0.002
Sequence number								
One primary only	Reference				Reference		Reference	
First of two or more	0.956 (0.897–1.018)	0.161			0.855 (0.792–0.923)	< 0.001	0.749 (0.693–0.809)	< 0.001
Chemotherapy								
Yes	Reference		Reference		Reference		Reference	
No	1.204 (1.122–1.292)	< 0.001	1.039 (0.966–1.118)	0.304	1.073 (0.986–1.168)	0.100		
Radiation								
Yes	Reference		Reference		Reference		Reference	
No	1.256 (1.117–1.412)	< 0.001	1.022 (0.906–1.153)	0.721	1.169 (1.023–1.336)	0.022	1.054 (0.919–1.209)	0.448
Marital status								
Married	Reference		Reference		Reference		Reference	
Unmarried	1.404 (1.318–1.497)	< 0.001	1.420 (1.329–1.517)	< 0.001	1.391 (1.292–1.498)	< 0.001	1.388 (1.286–1.498)	< 0.001

HR, hazard ratio; CI, confidence interval

### Survival analysis after 1:1 PSM

**Fig. 4:**
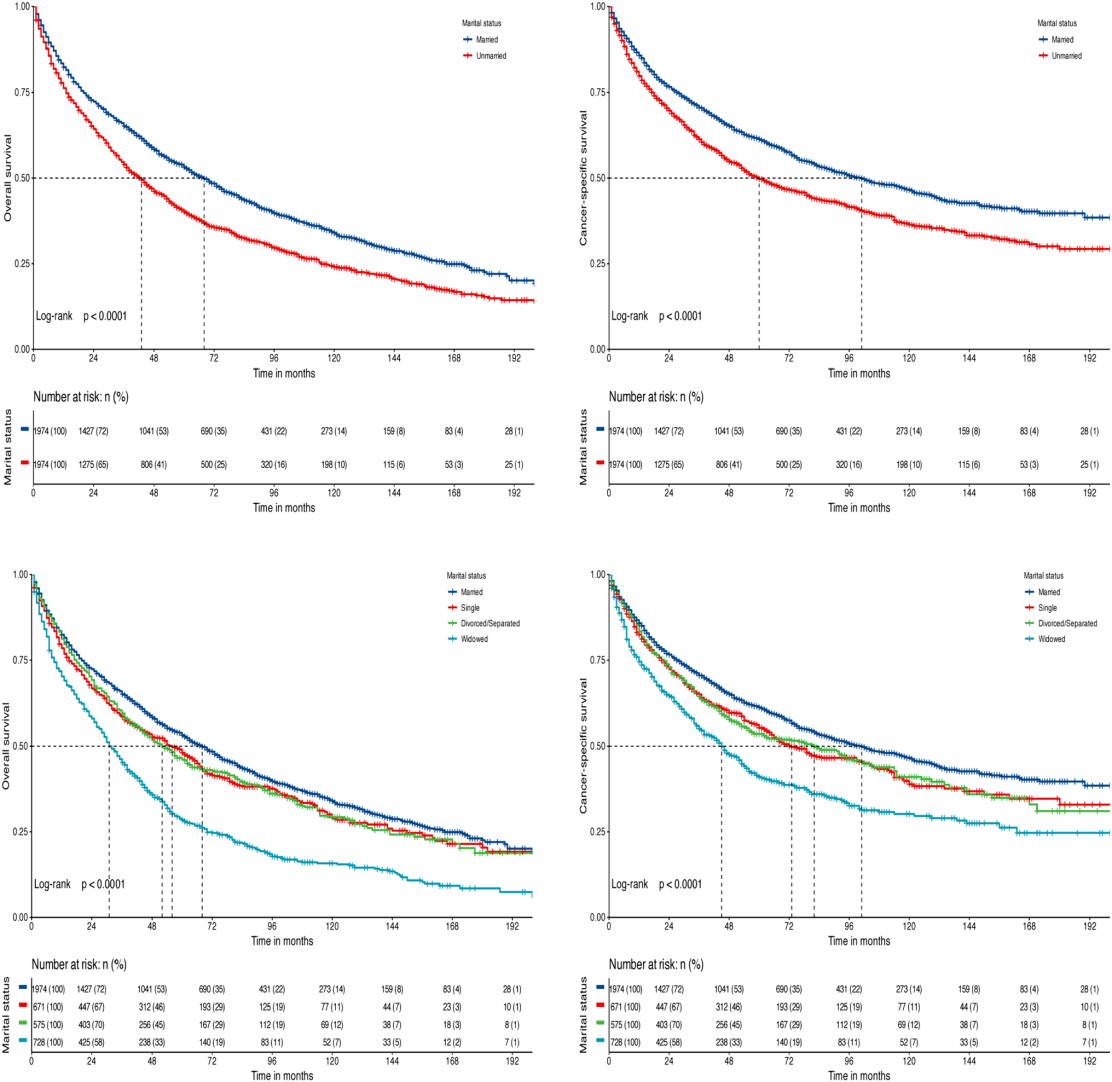
Kaplan–Meier curves present the overall survival and cancer-specific survival of patients with mantle cell lymphoma stratified by marital status after propensity score matching

Although the survival difference between married and unmarried patients is less pronounced after PSM, the survival of unmarried patients was still worse. The 5-year OS and CSS rates for unmarried patients were 40.2% (95% CI 38.0–42.5%) and 49.7% (95% CI 47.4–52.2%), respectively. Further survival analysis among unmarried patients revealed that the 5-year OS and CSS rates for singles were 48.1% (95% CI 44.3–52.1%) and 55.4% (95% CI 51.5–59.5%), respectively. Divorced/separated individuals exhibited the 5-year OS and CSS rates of 46.1% (95% CI 42.1–50.5%) and 53.6% (95% CI 49.4–58.1%), respectively. Widowed individuals showed 5-year OS and CSS rates of 28.5% (95% CI 25.3–32.1%) and 41.0% (95% CI 37.1–45.2%), respectively, as shown in Fig. 4.

To validate independent prognostic factors associated with MCL patients after PSM, we conducted both univariate and multivariate analyses. Being unmarried remained an independent risk factor influencing OS (HR 1.381, 95% CI 1.278–1.493, *p* < 0.001) and CSS (HR 1.377, 95% CI 1.258–1.507, *p* < 0.001) (Table 3).

**Table 3:** Univariate and multivariate analyses of overall survival and cancer-specific survival in patients with mantle cell lymphoma after propensity score matching

Characteristics	Overall survival				Cancer-specific survival			
	Univariate analysis	*p*-value	Multivariate analysis	*p*-value	Univariate analysis	*p*-value	Multivariate analysis	*p*-value
	HR (95% CI)		HR (95% CI)		HR (95% CI)		HR (95% CI)	
Sex								
Male	Reference		Reference		Reference		Reference	
Female	0.739 (0.682–0.801)	< 0.001	0.703 (0.647–0.763)	< 0.001	0.730 (0.664–0.802)	< 0.001	0.716 (0.651–0.788)	< 0.001
Age (year)								
≤ 50	Reference		Reference		Reference		Reference	
50–59	1.537 (1.233–1.916)	< 0.001	1.616 (1.296–2.014)	< 0.001	1.423 (1.115–1.817)	0.005	1.551 (1.213–1.982)	< 0.001
60–69	2.172 (1.755–2.689)	< 0.001	2.276 (1.838–2.819)	< 0.001	1.948 (1.538–2.469)	< 0.001	2.132 (1.682–2.704)	< 0.001
≥ 70	4.181 (3.412–5.124)	< 0.001	4.379 (3.569–5.372)	< 0.001	3.368 (2.688–4.218)	< 0.001	3.853 (3.069–4.837)	< 0.001
Year of diagnosis								
2000–2004	Reference		Reference		Reference		Reference	
2005–2009	0.926 (0.840–1.020)	0.119	0.879 (0.797–0.970)	0.010	0.914 (0.817–1.023)	0.119	0.852 (0.761–0.954)	0.006
2010–2015	0.700 (0.632–0.776)	< 0.001	0.755 (0.680–0.837)	< 0.001	0.679 (0.603–0.764)	< 0.001	0.704 (0.625–0.793)	< 0.001
Race/ethnicity								
Hispanic	Reference				Reference			
Non-Hispanic White	1.054 (0.915–1.214)	0.467			0.930 (0.769–1.088)	0.365	0.889 (0.760–1.040)	0.142
Non-Hispanic Black	0.914 (0.740–1.130)	0.407			0.777 (0.609–0.992)	0.043	0.835 (0.654–1.066)	0.148
Other	0.955 (0.748–1.219)	0.710			0.962 (0.736–1.256)	0.774	1.004 (0.768–1.312)	0.977
Stage								
Limited	Reference		Reference		Reference		Reference	
Advanced	1.177 (1.086–1.275)	< 0.001	1.205 (1.079–1.346)	0.001	1.393 (1.223–1.586)	< 0.001	1.446 (1.267–1.651)	< 0.001
Primary Site								
Extranodal	Reference				Reference			
Nodal	1.132 (1.021–1.256)	0.019	0.938 (0.840–1.047)	0.254	1.244 (1.092–1.419)	0.001	1.179 (1.031–1.347)	0.016
Sequence number								
One primary only	Reference				Reference		Reference	
First of two or more	1.021 (0.941–1.108)	0.617			0.851 (0.771–0.939)	0.001	0.736 (0.666–0.813)	< 0.001
Chemotherapy								
Yes	Reference		Reference		Reference			
No	1.134 (1.038–1.239)	0.005	1.025 (0.934–1.124)	0.605	1.021 (0.919–1.134)	0.705		
Radiation								
Yes	Reference		Reference		Reference			
No	1.311 (1.119–1.535)	0.001	1.055 (0.896–1.242)	0.519	1.173 (0.982–1.402)	0.078		
Marital status								
Married	Reference		Reference		Reference		Reference	
Unmarried	1.355 (1.254–1.464)	< 0.001	1.381 (1.278–1.493)	< 0.001	1.343 (1.227–1.469)	< 0.001	1.377 (1.258–1.507)	< 0.001

HR, hazard ratio; CI, confidence interval

### Subgroup analysis of the impact of different marital status on survival after propensity score matching.

**Fig. 5:**
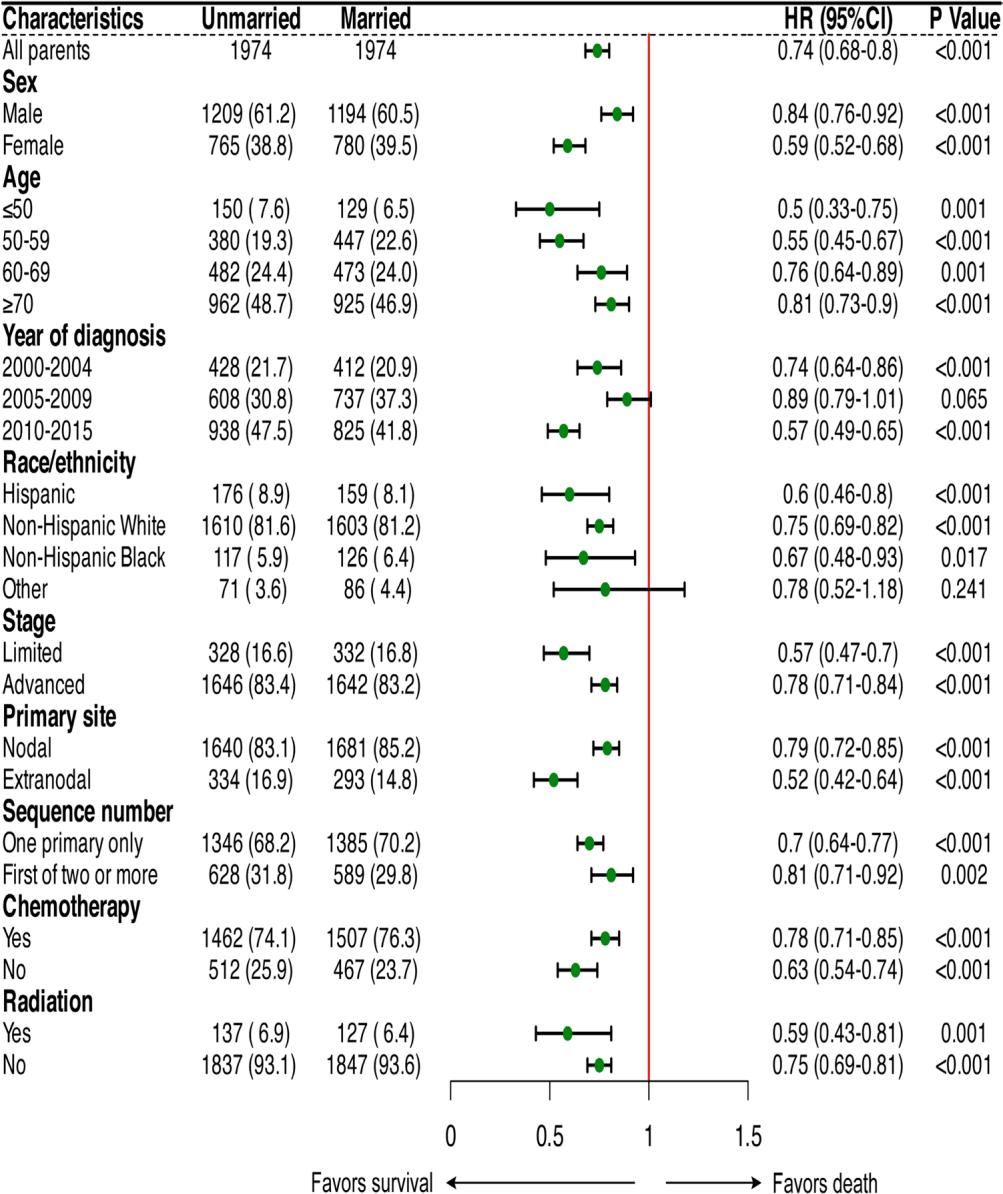
The forest plot presents a subgroup analysis of the impact of marital statuses on overall survival after propensity score matching. HR hazard ratio; CI confidence interval

We conducted subgroup analysis to further elucidate the impact of different marital status on survival outcomes across diverse subgroups. The results revealed that being married positively influenced survival in all subgroups. Despite the lack of significant differences in marital status for the diagnosis years 2005–2009 (*p* = 0.056) and other race/ethnicity (*p* = 0.241), the survival benefit persisted in these cases, as shown in Fig. 5.

### 1-, 5- and 10-year hazard models

An extended analysis of unmarried subgroup revealed an interesting phenomenon. Widowed patients showed inferior survival outcomes at 1-, 5-, and 10-year intervals compared to patients with other marital status. Particularly noteworthy, in the 10-year OS and CSS hazard model for widowed individuals, the risk of mortality was significantly higher, with the probability of the risk of overall and cancer-specific mortality increased by 1.7-fold and 1.6-fold, respectively (Table 4).

**Table 4:** 1-, 5- and 10-year hazard models of overall survival and cancer-specific survival based on different marital statuses in patients with mantle cell lymphoma

Hazard model	1-year OS		5-year OS		10-year OS		1-year CSS		5-year CSS		10-year CSS	
Marital status	HR (95% CI)	*p*-value	HR (95% CI)	*p*-value	HR (95% CI)	*p*-value	HR (95% CI)	*p*-value	HR (95% CI)	*p*-value	HR (95% CI)	*p*-value
Married	Reference		Reference		Reference		Reference		Reference		Reference	
Single	0.966 (0.795–1.174)	0.732	1.111 (0.980–1.260)	0.098	1.132 (1.011–1.267)	0.031	0.955 (0.768–1.187)	0.679	1.135 (0.986–1.305)	0.076	1.173 (1.032–1.333)	0.014
Divorced/separated	0.930 (0.749–1.155)	0.511	1.052 (0.922–1.199)	0.450	1.146 (1.017–1.292)	0.024	1.041 (0.823–1.316)	0.734	1.089 (0.942–1.259)	0.248	1.180 (1.031–1.351)	0.016
Widowed	1.170 (0.987–1.387)	0.070	1.393 (1.248–1.553)	< 0.001	1.722 (1.557–1.905)	< 0.001	1.225 (1.015–1.479)	0.032	1.291 (1.138–1.463)	< 0.001	1.584 (1.407–1.783)	< 0.001

HR, hazard ratio; CI, confidence interval; OS, overall survival; CSS, cancer-specific survival

## Discussion

MCL represents an incurable and heterogeneous form of lymphoma, exhibiting a 5-year survival rate of 52.5%(Kamel Mohamed et al. 2020). The clinical factors related to the prognosis of MCL include age, sex, stage, physical status, lactate dehydrogenase (LDH), white blood cell count, and Ki-67 index (Wu et al. 2020; Jain et al. 2022). However, existing studies have unexplored the relationship between marital status and survival outcomes in MCL.

Based on our study, marital status significantly impacted OS and CSS. Specifically, widowed patients had lower 5-year OS and CSS rates compared to patients with other marital status. Conversely, married patients demonstrated superior OS and CSS rates compared to patients with other marital statuses. Consequently, marital status was identified as an independent risk factor for survival outcomes in MCL patients.

Numerous studies have confirmed the impact of marital status on cancer survival (Li et al. 2015; Jin et al. 2016; Wang et al. 2017; Xing et al. 2021; Wu et al. 2022). Aizer, et al. found that widowed patients faced a greater risk of developing metastatic cancer, receiving unbalanced treatment, and experiencing death linked to their cancer when compared to married patients (Aizer et al. 2013). This study is the first to analyze the impact of marital status on OS and CSS in patients with MCL based on the SEER database, which has important implications for clinicians to more comprehensively assess the prognosis of patients with MCL.

The impact of marital status on the survival of cancer patients can be explained from the perspective of social psychology. Cancer patients have more serious psychological distress than other patients. Married patients showed less depression, anxiety, and distress after a cancer diagnosis by having their spouse help combat negative emotional distress and receive strong social support from friends and family (Goldzweig et al. 2010; Kaiser et al. 2010). There was a strong association between psychological distress and poor adherence to treatment, and patients experiencing depression were found to be three times more likely to fail to comply with medication recommendations compared to those who did not have depression (DiMatteo et al. 2000). McCowan, et al. found that breast cancer patients with high adherence to tamoxifen treatment had a lower recurrence rate of 8.95% and a lower mortality rate of 8.65%(McCowan et al. 2013). Patients who are married often have a lower risk of developing major depression (Weissman et al. 1996). Goodwin, et al. concluded that breast patients diagnosed with depression who received nondefinitive treatment had greater risk and worse survival than those who received definitive treatment (Goodwin et al. 2004).

An abnormal cortisol circadian rhythm can predict early death of cancer patients, while restraint in natural killer cell quantity and function might signify speedy disease progression (Sephton et al. 2000, 2013). Studies have shown that better quality social support is associated with healthier neuroendocrine function, which has significant implications for cancer prognosis (Turner-Cobb et al. 2000). Additionally, it should not be ignored that married people have a lower risk of alcohol abuse and smoking than those with other marital status (Leonard and Rothbard 1999; Lindström 2010), which could be advantageous for the well-being of cancer patients.

As a population-based retrospective study, there are inevitably some limitations. First of all, the SEER database lacks detailed information related to the treatment of MCL patients, such as the regimen of chemotherapy, the application of targeted drugs, and the evaluation of efficacy. Secondly, important features related to MCL prognoses, such as ECOG score, LDH, ki-67 index, and white blood cell count, were lacking. Finally, we hypothesized that psychosocial and treatment adherence factors were responsible for the poor survival of widowed patients, but the SEER database lacked records of psychological tests, mental status, and treatment adherence assessments of MCL patients. Additionally, some confounding variables that affect the outcome of patients with MCL, such as smoking and alcohol abuse, were not available from the SEER database. This may lead to some bias in the analysis results, and further research is necessary to verify it.

## Conclusion

The first study to analyze the relationship between marital status at diagnosis and survival in patients with MCL, the results of this study demonstrate that marital status at diagnosis is an independent prognostic factor for patients with MCL, with widowed patients showing worse OS and CSS than those who are married, single, and divorced/separated. It is important to note that adequate psychological and social support for widowed patients can help improve outcomes for such patients.

### Supplementary Information

Below is the link to the electronic supplementary material.

Supplementary file 1 (DOCX 458 kb)

## Data Availability

Data from the SEER can be obtained upon request to the SEER. This data can be found here: https://seer.cancer.gov/data/.
