# Supplementary material for: The influence of marital status at diagnosis on survival of adult patients with mantle cell lymphoma
Source: J Cancer Res Clin Oncol. 2024 Mar 11;150(3):120. doi: 10.1007/s00432-024-05647-z (PMC10927831; doi:10.1007/s00432-024-05647-z)
Supplement: Supplementary file 1 — (DOCX 458 kb) [file 432_2024_5647_MOESM1_ESM.docx]

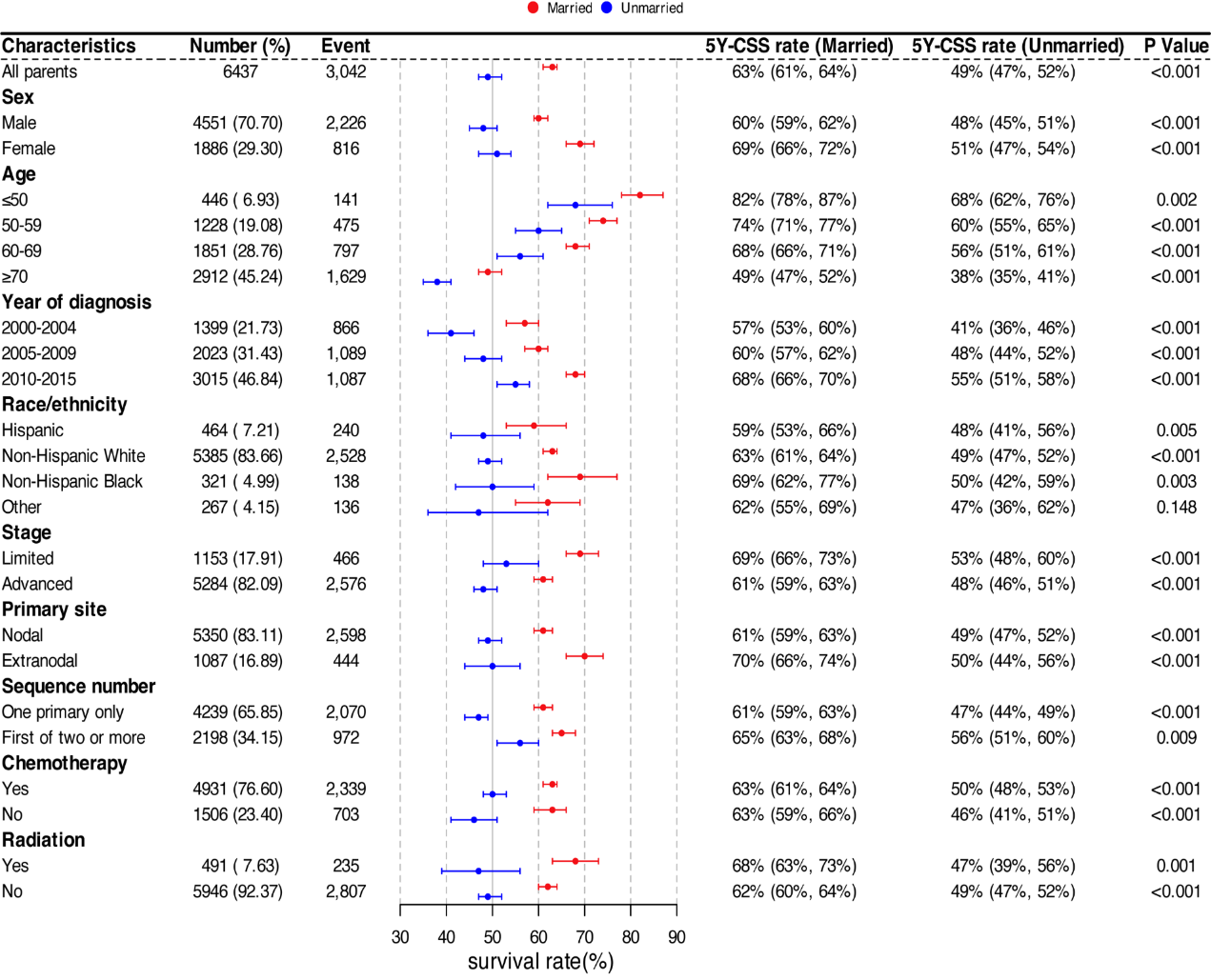
**Supplementary Fig. S1** The 5-year cancer-specific survival rates among different marital statuses within each subgroup. 5Y-CSS 5-year cancer-specific survival.
